# *In situ* Raman spectroscopy and machine learning unveil biomolecular alterations in invasive breast cancer

**DOI:** 10.1117/1.JBO.28.3.036009

**Published:** 2023-03-30

**Authors:** Sandryne David, Trang Tran, Frédérick Dallaire, Guillaume Sheehy, Feryel Azzi, Dominique Trudel, Francine Tremblay, Atilla Omeroglu, Frédéric Leblond, Sarkis Meterissian

**Affiliations:** a Polytechnique Montréal, Department of Engineering Physics, Montreal, Quebec, Canada; b Centre de recherche du Centre hospitalier de l’Université de Montréal, Montreal, Quebec, Canada; c Institut du cancer de Montréal, Montreal, Quebec, Canada; d Université de Montréal, Department of Pathology and Cellular Biology, Montreal, Quebec, Canada; e McGill University Health Center, Department of Surgery, Montreal, Quebec, Canada; f McGill University Health Center, Department of Pathology, Montreal, Quebec, Canada

**Keywords:** Raman spectroscopy, breast cancer, breast-conserving surgery, machine learning, tissue optics, support vector machines, biochemistry

## Abstract

**Significance:**

As many as 60% of patients with early stage breast cancer undergo breast-conserving surgery. Of those, 20% to 35% need a second surgery because of incomplete resection of the lesions. A technology allowing *in situ* detection of cancer could reduce re-excision procedure rates and improve patient survival.

**Aim:**

Raman spectroscopy was used to measure the spectral fingerprint of normal breast and cancer tissue *ex-vivo*. The aim was to build a machine learning model and to identify the biomolecular bands that allow one to detect invasive breast cancer.

**Approach:**

The system was used to interrogate specimens from 20 patients undergoing lumpectomy, mastectomy, or breast reduction surgery. This resulted in 238 *ex-vivo* measurements spatially registered with standard histology classifying tissue as cancer, normal, or fat. A technique based on support vector machines led to the development of predictive models, and their performance was quantified using a receiver-operating-characteristic analysis.

**Results:**

Raman spectroscopy combined with machine learning detected normal breast from ductal or lobular invasive cancer with a sensitivity of 93% and a specificity of 95%. This was achieved using a model based on only two spectral bands, including the peaks associated with C–C stretching of proteins around $940\;{\mathrm{cm}}^{-1}$ and the symmetric ring breathing at $1004\;{\mathrm{cm}}^{-1}$ associated with phenylalanine.

**Conclusions:**

Detection of cancer on the margins of surgically resected breast specimen is feasible with Raman spectroscopy.

## 1 Introduction

Breast cancer is the most diagnosed cancer worldwide. It accounted for 1 in 8 cancer diagnoses and 685,000 deaths in 2020.^1^^,^^2^ It is the leading cause of cancer mortality in women and may affect men in rare cases.^1^^,^^3^ Early diagnosis and effective treatment are critical factors for survival.^4^ Ductal carcinoma is the most common type of breast cancer, and it originates in epithelial cells lining the interior of the ducts that carry milk from glands to the nipple. Lobular carcinoma, the second most common breast cancer type, originates from cells within the lobules, which are groups of glands connected to the duct. These carcinomas may be *in situ* or invasive.^5^ *In situ* cancers have not grown into surrounding tissues, whereas invasive cancer cells have initiated molecular processes allowing them to spread beyond the tissue of origin.^5^ There are two major types of *in situ* breast cancers: ductal carcinoma *in situ* and lobular carcinoma *in situ*.^6^

The most common breast imaging modality is x-ray mammography, generally followed by a biopsy and a histopathology analysis to assess cancer type.^7^ Adjuvant therapies are available to treat breast cancer (e.g., immunotherapy and chemotherapy) but standard of care involves surgical excision of the tumor followed by radiotherapy. Surgical options include breast-conserving surgery, mastectomy, and lymph nodes resection. Breast-conserving surgery is the recommended treatment for early stage patients. The aim of this procedure is to excise cancer with a margin of normal tissue surrounding the tumor. For early stage patients, long-term outcomes of breast-conserving surgery are equivalent to mastectomy if clear margins are obtained.^8^

The success of breast-conserving surgery therefore involves the complete removal of malignant tissue, including a circumferential margin of normal tissue surrounding the tumor. Because the presence of cancer cells on the margin is associated with a greater risk of cancer recurrence, specimen margins assessment by a pathologist can be required during a procedure to inform surgeons if more tissue needs to be removed. However, despite intraoperative pathological evaluation and radiological analyses, for up to 20% to 35% of cases positive margins are missed, resulting in the need for a second surgery.^9^^,^^10^ Re-excision procedures lead to additional costs, patient anxiety, and an increased risk of post-surgical complications, highlighting the need for new intraoperative surgical guidance techniques.

Multiple emerging imaging techniques were developed for live *in situ* intra-operative breast tissue examination. Ultra-sound imaging was used to visualize structural tissue features and has proved an effective, rapid, and low-cost margins assessment technique.^11^ Radiofrequency spectroscopy, which is based on differences in scattering, reflectance, and absorbance, was designed for hand-held use and has shown promises for intraoperative margins detection.^12^ Impedance spectroscopy imaging, which relies on detecting dielectric tissue properties, was also developed as a portable handheld imaging device allowing rapid scanning.^13^ Although these techniques showed promise, breast cancer studies conducted using them showed levels of sensitivity and specificity that may limit their potential to reduce re-excision rates following breast-conserving surgery. Fiber-optics systems were also developed for intra-operative use relying on diffuse reflectance spectroscopy^14^ and optical coherence tomography.^15^ Other methods were used for *ex vivo* whole-specimen interrogation. Photoacoustic tomography used fat and hemoglobin as sources of contrast, leading to high-chemical selectivity but limited specificity to distinguish connective tissue and tumor.^16^ Spatial frequency domain imaging identified pathology subtypes based on the optical contrast provided by elastic scattering^17^ while other studies used either fluorescence lifetime imaging^18^ or tissue autofluorescence combined with diffuse reflectance spectroscopy.^19^

Microscopy techniques were developed for breast tissue studies with an emphasis on oncology. Those methods had a more limited field of view when compared to the *in situ* and *ex vivo* whole-specimen methods, but they were able to reveal more subtle structural and/or biochemical at high spatial resolution, with the enticing prospect to replace—or at least complement—standard histopathology analyses relying on staining techniques using dyes such as hematoxylin and eosin (H&E). A microscopic imaging approach based on ultra-violet surface excitation imaged tissue fluorophores without the requirement for tissue fixing, embedding, and sectioning.^20^ Light-sheet microscopy provided rapid, non-destructive, slide-free 2D and 3D imaging with the same level of detail as standard histopathology methods.^21^ Other microscopy techniques relied on non-linear optical contrast to provide high resolution molecular images.^22^^–^^24^

Raman spectroscopy showed potential for breast cancer detection. Macroscopic inelastic scattering point measurements^25^ and Raman micro-spectroscopy^26^^,^^27^ in biopsy specimens demonstrated the technique can be used to detect cancer. Raman spectroscopy studies by our group also showed potential for *in situ* intraoperative cancer detection of other pathologies, including brain,^28^^–^^30^ ovaries,^31^ and prostate.^32^^–^^35^

**Fig. 1:**
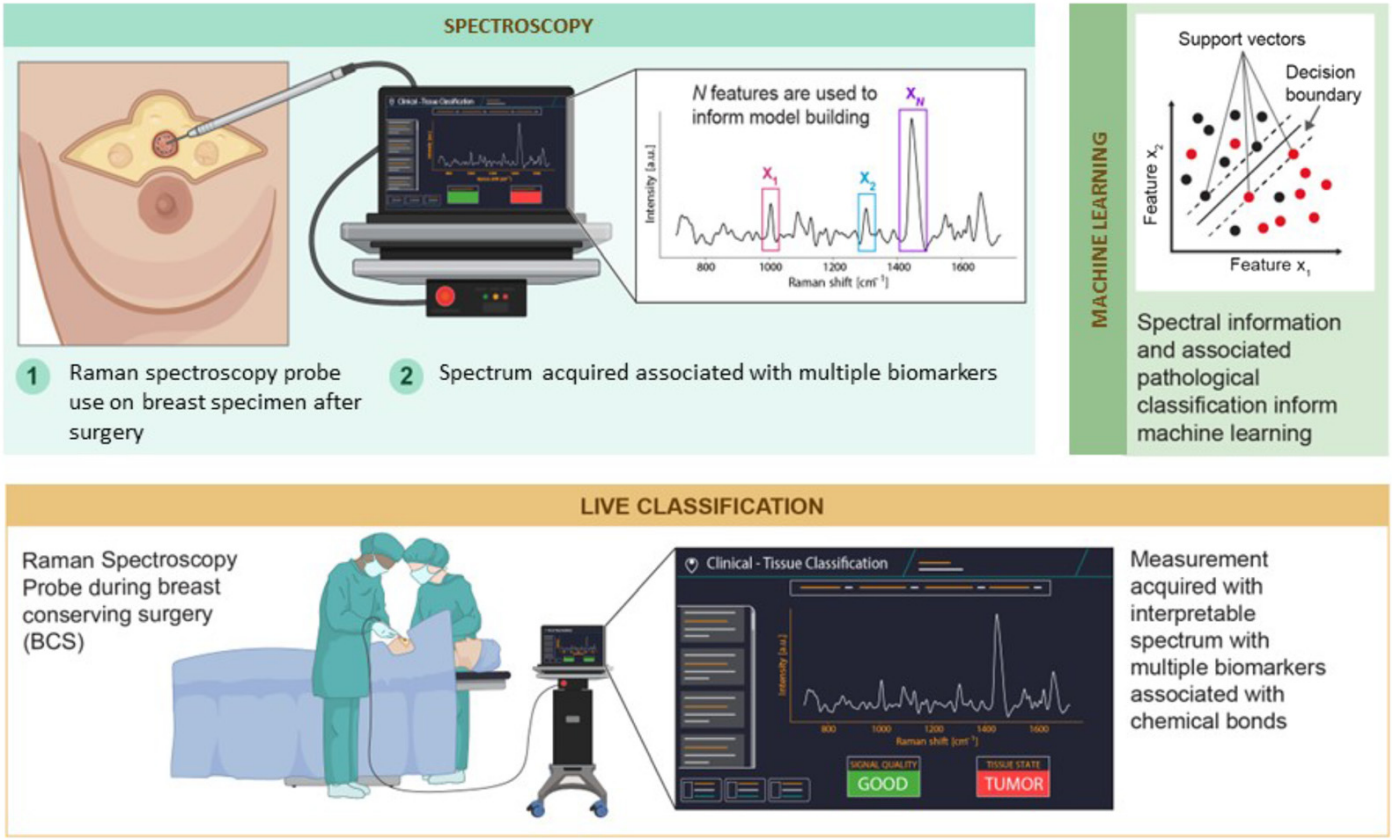
Schematic representation of the Raman spectroscopy hand-held probe workflow developed for surgical-guidance during breast-conserving surgery. *Ex vivo* spectroscopic measurements were made on breast surgery specimen to train machine learning models. The machine learning techniques selected the biomolecular spectral features with the most contrast between cancer and normal breast, and those features were used to train and test the cancer detection predictive models. The intent is to use those models for live *in situ* cancer detection during breast-conserving surgery.

This work presents the results of a study that led to the acquisition of an *ex vivo* Raman spectroscopy dataset in human breast, including normal tissue and invasive ductal or lobular carcinoma. The study was designed to allow machine learning models to be developed based on the intrinsic macroscopic optical signature of multiple forms of tissue to distinguish invasive breast cancer from normal breast. The study also provided an assessment whether the classification models can shed light on specific biomolecular features of breast cancer using Raman spectroscopy, and whether the approach showed potential for surgical guidance in breast-conserving surgery to reduce re-excision procedure rates. A Raman spectroscopy single point probe system was used on fresh *ex vivo* specimens to train the cancer detection models but the intent is to use those same models for live *in situ* cancer detection during surgical procedures (Fig. 1).

## 2 Methods

### 2.1 Raman Spectroscopy System

Measurements were made using an intra-operative hand-held single-point Raman spectroscopy probe system manufactured by the company Reveal Surgical (Montreal, Canada). The fiber-optics probe design of similar systems was presented elsewhere.^30^^,^^36^ Briefly, it integrates ten fiber-optics of 100-μm diameter core. Of these fibers, nine were used for light detection and a central fiber was used for tissue excitation. A band-pass filter covered the tip of the excitation fiber, allowing illumination centered around 785 nm, and a high-pass filter was used for the collection fibers. The probe was connected to a 785-nm laser and a spectrometer with high sensitivity at wavelengths ranging from 800 to 900 nm, with an average resolution of $1.8\;{\mathrm{cm}}^{-1}$ across the spectral detection domain. A converging lens at the tip of the probe ensured contact measurements interrogated a spot size of diameter ∼0.5  mm. The system was controlled by a proprietary software (Reveal Surgical, Montreal, Canada) that allowed acquisition parameters to be set by the user, including laser power, exposure time per spectrum, and number of repeated measurements (i.e., accumulations) at each point.

### 2.2 Patients Selection

About 20 patients were recruited for this study. Of those, 19 underwent breast surgery (lumpectomy or mastectomy) following a diagnosis of invasive breast cancer (lobular or ductal) and one patient underwent breast reduction surgery. For that patient, spectroscopic measurements were made from a breast in which no tumor was radiologically detectable. However, the patient was diagnosed with breast cancer associated with a tumor detected in the contralateral breast. All patients recruited in the study were undergoing breast surgery for the first time, did not have neoadjuvant therapy, had a cancer grade inferior to 4, and had a tumor larger than 1 cm.

Patient specimens were utilized to build an *ex vivo* dataset of Raman spectroscopy measurements combined with histopathological and clinical data. Informed consent was obtained before the patient underwent surgery (McGill University Health Center Ethics Committees, approval number 2021-5997). Clinical data available included age, tumor type, tumor size, and Nottingham histologic score. Patient demographic details are provided in Table 1 (see also Table 3 for demographic details on each patients). The breast reduction surgery patient was recruited with the intention to acquire more measurements in healthy tissue to ensure a more balanced dataset.

**Table 1:** Clinical and pathological characteristics of all patients undergoing breast surgery (std: standard deviation; NA: not available; and IQR: interquartile range).

# Patient	20
Median age (years ± IQR)	67 ± 14
Type of surgery	
Breast-conserving surgery	14
Mastectomy	5
Breast reduction surgery	1
# of patients per tumor type	
Invasive ductal carcinoma	13
Invasive lobular carcinoma	3
Invasive mammary not otherwise specified (NOS)	2
No tumor	1
NA	1
Tumor size average (cm)	2.5
Nottingham histologic score # patient	
1	2
2	10
3	6
No tumor	1
NA	1

### 2.3 Specimen Handling and *ex vivo* Spectroscopic Measurements

**Fig. 2:**
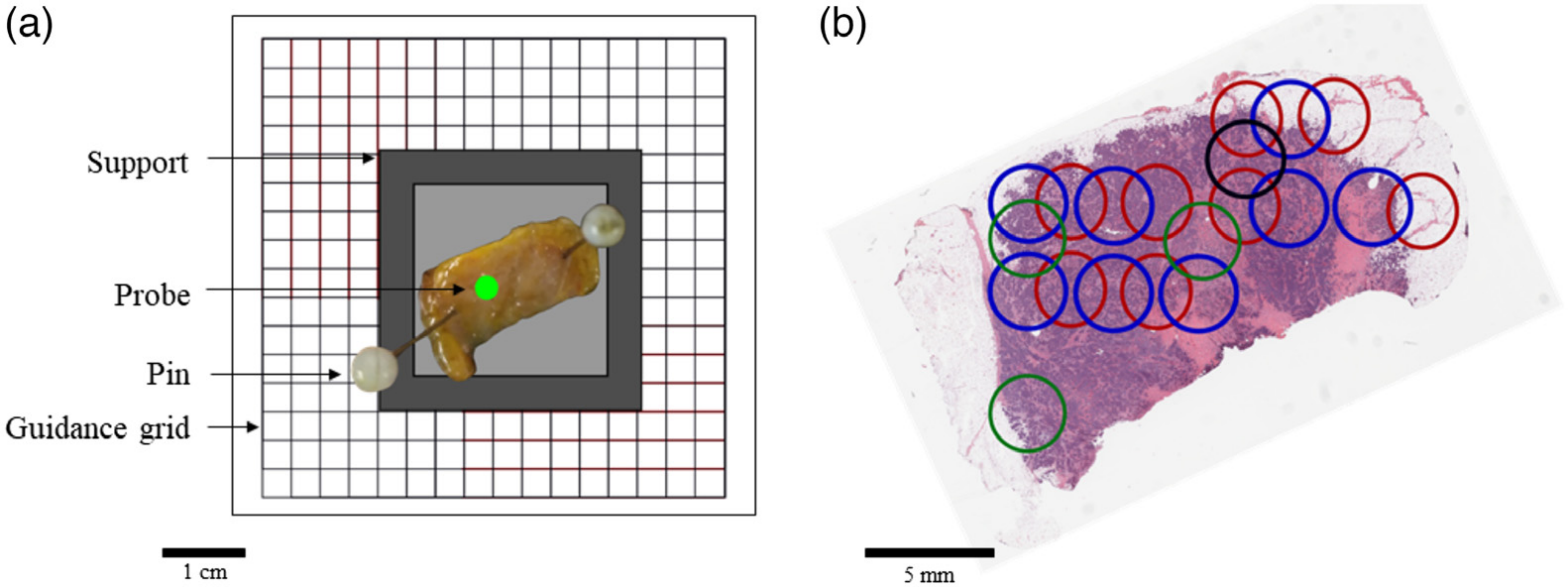
Spatial registration of Raman spectroscopy measurements with histopathology: (a) Representation of the technique where a fresh specimen was initially pinned to a cardboard support placed on a guiding template in the form of a grid. The circle superposed with the specimen represents the position of the probe above the grid. (b) End result of the methodology leading to the spatial registration of all spectroscopic measurements with an H&E-stained section. Different colors were used for some of the circles to improve visualization in cases when they overlapped.

Twenty breast specimens (one per patient) were extracted by the surgeons (either S.M. or F.T.). The specimens were weighed, measured, and marked with ink, following institutional standards. They were then cut in serial slices of ∼5-mm thickness. For each specimen, two smaller samples were cut from two different slices. Those samples were selected by the pathologist (A.O.) based on visual inspection with the objective of having one sample containing cancer cells and one sample mostly composed of healthy tissue. The samples were fixed on a cardboard support using two pins and a photograph was taken. The support was placed on a grid template and the Raman spectroscopy probe was fixed above the center of the grid [Fig. 2(a)]. Before each measurement, the cardboard support was moved to a different location on the template. The xy position with respect to the lower right corner of the cardboard support was recorded for each measurement.

The laser power of the system was set at a value that resulted in 100 mW being delivered to the tissue surface. The number of repeat measurements per point (accumulations) was fixed at N=10 and the exposure time per spectrum ranged from 0.1 to 4 s, including dark noise (background) measurements made with the laser off. The exposure time was adjusted to ensure a raw signal intensity above 60% of the sensor dynamical range while avoiding saturation. All measurements were acquired within a one-hour timeframe after surgical excision and the size of the samples was limited to $∼2\;{\mathrm{cm}}^{2}$ to preserve the integrity of the specimen and the tumor for margin evaluation, as part of the regular post-surgery clinical diagnostic workflow. The size limitation and the time constrain limited the number of independent Raman measurements to—on average—10 per sample (i.e., 20 per patient). After spectroscopic measurements, the samples were fixed in formalin, embedded in paraffin, and sectioned in 3 to 10  μm histological sections. They were then stained with H&E as per institutional standards, resulting in one stained image for each sample.

### 2.4 Registration with Optical Measurements and Histology LABELS

A four-step methodology was developed leading to spatial registration of all Raman spectroscopy measurements with corresponding locations on the H&E images using the Inkscape software (Inkscape’s Contributors). First, macroscopic photographs of the breast sample on the cardboard support [Fig. 2(a)] were taken. Second, the H&E images were superposed to the photograph and rotated to ensure the contours of the stained tissue sections matched as much as possible the contours seen on the photograph. Irregularities in the specimen shape were used as geometrical landmarks guiding this spatial registration process. Visible structures, including blood vessels and regions with high adipose content, were also used as tissue-based fiducial markers to ensure an accurate superposition of the photographs with the H&E images. Third, a numerical version of the registration grid was superposed to the H&E image, and it was used to pinpoint the location of every spectroscopic measurement. Fourth, each measurement location was annotated with a 3-mm diameter circle. The size of that circle was set to be two times larger when compared to the tip of the probe, accounting for potential spatial registration inaccuracies associated with sample deformations. As exemplified in Fig. 2(b), this led to images where colored circles indicated measurement locations superposed with the H&E images. Different colors were used for some of the circles to improve visualization in cases when they overlapped.

The resulting images were analyzed by pathologists (D.T., F.A.) to determine which types of cells were contained within each circled region. Cells were classified into three categories: (1) cancer (tumor cells, tumor stroma, or necrosis); (2) normal: either normal breast (connective tissue, stroma, fibroblast, and collagen) or breast parenchyma (ducts and lobules); (3) fat (adipose cells). The number of cells from each category was counted and the percentage of cancer, normal, or fat cells within each circle were computed. A measurement was labeled as cancer if the percentage of cancer cells was equal or superior to 80%, normal if the percentage of normal cells was equal or superior to 80%, and fat if the percentage of adipose cells was equal or superior to 70%. All measurements that did not fit into one of the three classes were excluded.

### 2.5 Data Processing and Labeling

The data processing steps preceding the production of machine learning models led to the extraction of the inelastic scattering signature, for each measurement, from background contributions, including intrinsic tissue fluorescence.^37^ Spectral pre-processing included the following steps: (1) averaging of the N=10 repeat spectra to increase overall signal-to-noise ratio, (2) averaging and subtraction of background spectra acquired with the laser turned off between measurements, (3) normalization with a NIST Raman standard (SRM 2214) to correct for the instrument response, (4) x-axis (wavenumber shift) calibration based on a Raman spectrum acquired on polycarbonate, (5) removal of background signals using the custom background removal algorithm BubbleFill,^38^ and (6) standard normal variate (SNV) normalization.

A quantitative quality factor metric (between 0 and 1) was then computed from each resulting spectrum. It provided a statistical assessment of the likelihood the SNV-normalized signal is associated with tissue Raman peaks or stochastic noise.^39^ Spectra with a quality factor metric inferior to 0.6 were excluded as they did not always clearly show all the ubiquitous Raman peaks encountered in biological tissue (e.g., amide bands for protein-rich tissue). All spectra were then visually inspected individually to qualitatively identify any glaring labeling errors here, mostly based on the fact that adipose tissue Raman spectra have spectral features that are dramatically different when compared to protein-rich tissue. For example, pure fat does not contain phenylalanine and as a result does not present a visually detectable peak at $1004\;{\mathrm{cm}}^{-1}$. Spectra labeled as cancer or normal that were associated with clearly identifiable fat spectra were removed from the dataset, as well as spectra identified as fat that did not show the expected biomolecular features associated with proteins. All remaining spectra were then plotted along with the standard deviation for each of the following groups: normal, cancer, and fat. The average spectrum associated with the normal breast measurements of the breast reduction surgery patient was also plotted against all other normal spectra and the cancer spectra (Fig. 7).

### 2.7 Machine Learning Workflow

A machine learning workflow was applied to develop three different models: cancer versus normal + fat (model A), cancer versus normal (model B), and cancer versus fat (model C). Due to the small size of the dataset and to minimize the risk of over-fitting the data, the first step of the machine learning workflow consisted of dimensionally reducing the number of features per spectra to <10 for each model. This dimensional reduction procedure was done using a linear support vector machine (SVM) with a regularization by Lasso regression, in which individual statistical weights were assigned to each feature. This statistical weight acted as a surrogate for feature relevance, in effect quantifying the ability of each feature to capture inter-class variations and allowed less important bands to be discarded.^40^^,^^41^ Features were then ranked according to their weight and boxplots were produced to show the statistical distribution of spectral intensity for all features that were retained to produce the machine learning models (Table 2 and Fig. 5).

**Table 2:** Raman peaks and main corresponding vibrational bonds associated with the Raman-predicted molecular tissue content associated with the spectra in Fig. 3. The bands associated with spectral features used as inputs to the classification models (Model A, Model B, and Model C) are identified in the last column. A tentative molecular assignment (specific molecules and families of biomolecules) based on literature findings is shown.^42^

Peak center (${\mathrm{cm}}^{-1}$)	Main vibrational modes	Tentative biomolecular assignment	Classification models
760	Ring breathing tryptophan	Proteins	—
785	Cytosine; U, T, C (ring breathing modes in the DNA/RNA bases)	DNA/RNA	—
937-942	C–C stretching	Proteins (collagen)	A, B
1004	C–C stretching	Proteins (phenylalanine, collagen)	A, B, C
1129	Acyl backbone in lipid	Lipids	B
1159	C–C/C–N stretching (proteins)	Proteins	B
1176	C–H bending tyrosine, cytosine, guanine	DNA/RNA	—
1208	Ring breathing modes of the DNA/RNA bases, phenylalanine	DNA/RNA, proteins (phenylalanine, amide III)	—
1246, 1266	Amide III	Proteins	—
1301–1304	C–H vibration	Lipids	C
1600	C═C	Proteins (phenylalanine)	—

C─H: carbon-hydrogen single bonds, C═C: carbon-carbon double bonds (unsaturated), C─C: carbon-carbon bonds, ${\mathrm{CH}}_{2}$: ethyl group, ${\mathrm{CH}}_{3}$: methyl group.

The classification models training process involved SVM with a linear kernel and a regularization parameter C. This hyperparameter, which varied between $10^{-3}$ and 1, controlled the penalty for errors in the training process and reduced the risk of overfitting the data. The SVM algorithm also considered the class imbalance for all datasets by setting a higher cost γ to misclassified spectra from the least populated class.^41^ A grid search method was used to optimize both hyperparameters C and γ, and a five-fold cross-validation procedure was performed to assess classification performances. The five-fold cross validation took the training dataset and randomly split it in five equal-size groups. Then, five models were created with the same hyperparameters. Each model was trained on four groups and tested on the remaining one. The average of the performance of the five classifiers performance was used to assess predictive performance.^43^ During the cross-validation phase, data from the same patient were never used in both the training and testing sets.

The technique returned, for each measurement, a posterior probability 0≤p≤1 that a measurement was classified within one of two classes. A receiver-operating-characteristic (ROC) curve was computed by comparing this posterior probability p with a parameter λ(0≤λ≤1). All observations associated by the classifier were assigned the label 1 when p≥λ or assigned the label 0 if otherwise. Different values of the parameter λ corresponded to different points of the ROC curve. Sensitivity and specificity were computed by comparing the label assigned by the model to the label given by the histopathology analysis for every measurement. Results were reported for the optimized hyperparameters only, yielding the highest area-under-curve (AUC) value. The final optimized model, with reported accuracy, sensitivity, and specificity, corresponded to the ROC curve point with the smallest distance to the upper left corner of the curve (x-axis: 1 − specificity, y-axis: sensitivity).

## 3 Results

### 3.1 Spectroscopic Measurements

**Fig. 3:**
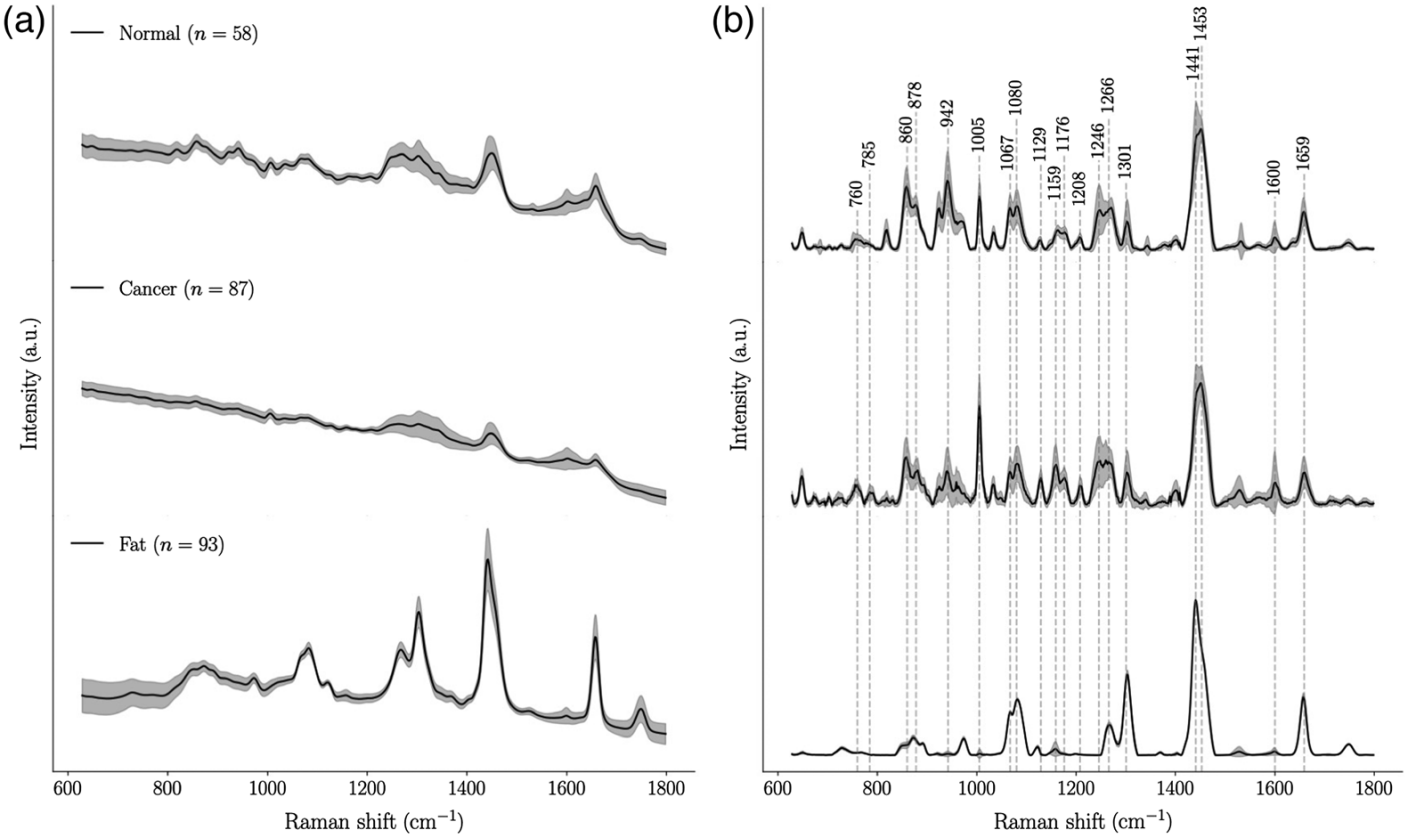
Spectra associated with normal, cancer, and fat measurements in breast tissue: (a) average spectroscopic measurements (raw data) for each tissue category along with the standard deviation for each spectral bin; (b) average Raman spectra and standard deviation with selected bands identified with their wavenumber value. All identified bands were used for the biochemical interpretation of the data (Table 2), and a subset of those bands was used for machine learning model development.

Application of the Raman spectroscopy measurement protocol resulted in 388 spectroscopic measurements with co-located histopathology analyses. Of those, 58 spectra were excluded because of visibly low spectral quality, non-tissue-related artifacts (e.g., from residual ambient light, Raman peaks from the cardboard substrate) or a quality factor lower than 0.6. Moreover, 58 measurements were excluded because they did not qualify for association with either the normal, cancer, or fat categories. Further, 34 spectra were excluded due to suspected labeling errors based on visual inspection comparing measured spectra with the known Raman signature of adipose tissue. Of those, 28 that were labeled as normal or cancer were excluded because the spectra were identical to fat spectra. The remaining six spectra, which were labeled as fat, were excluded because they showed clearly visible spectral features associated with protein-rich tissue. The final dataset was composed of 238 measurements: 58 were labeled as normal, 87 as cancer, and 93 as fat. The raw and processed average measurements for each tissue category, along with the signal variance associated with each spectral bin, are shown in Figs. 3(a) and 3(b), respectively. Selected bands of interest are identified in Fig. 3(b) and their biochemical interpretation is provided in Table 2.

### 3.2 Machine Learning Models and Biomolecular Predictions

**Fig. 4:**
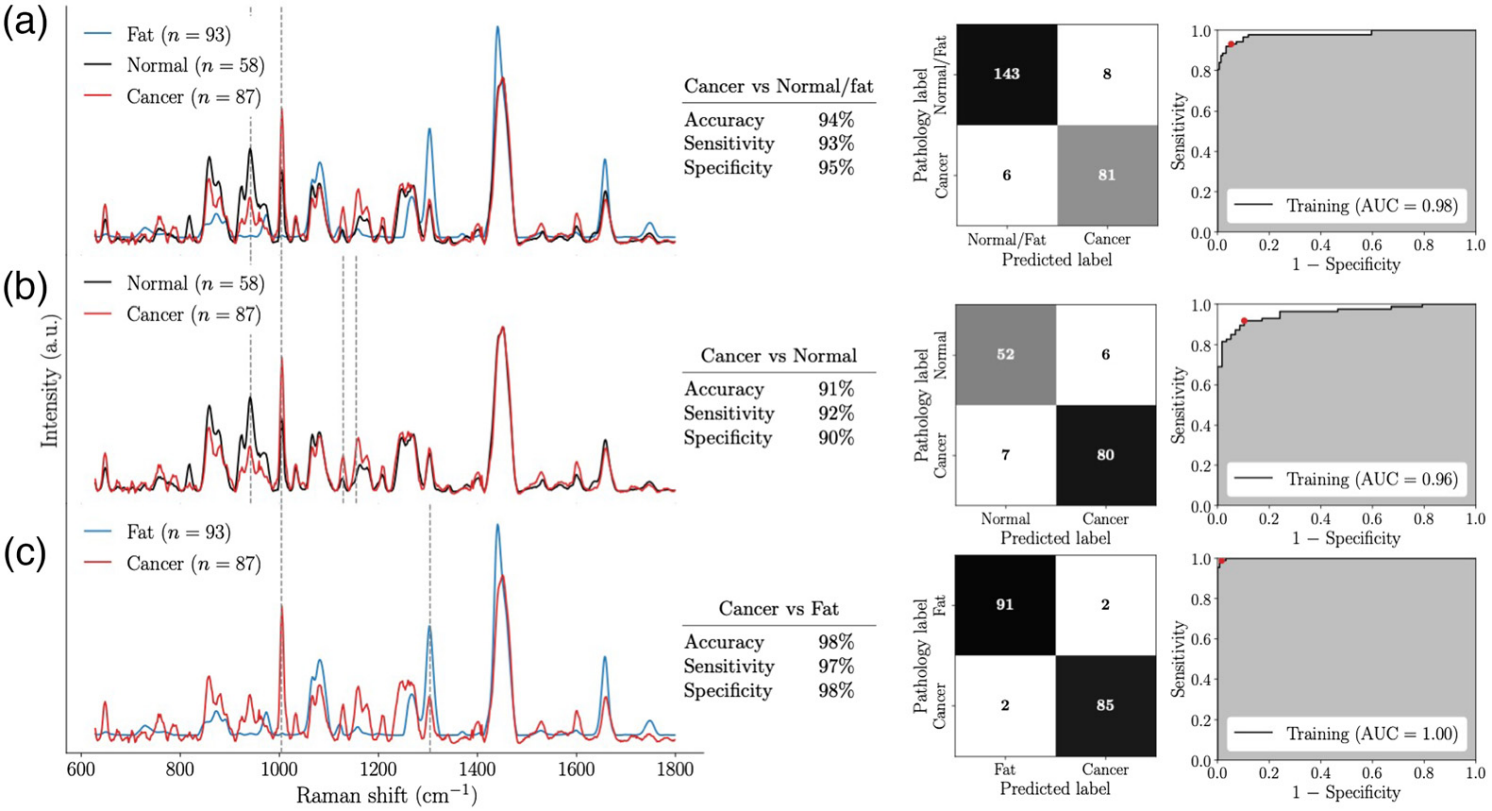
Average Raman spectra and spectral features (dotted lines) used by the classification models. Classification results are expressed in term of accuracy, sensitivity, and specificity. Confusion matrices and ROC curves are also shown for the three models: (a) Model A: cancer versus non-cancer (normal + fat); (b) Model B: cancer versus normal, and (c) Model C: cancer versus fat.

A classification model (Model A) was trained to distinguish between the cancer and non-cancer (fat + normal) spectra, from 238 measurements (87 cancer spectra, 58 normal spectra, and 93 fat spectra). This yielded an accuracy of 94%, a sensitivity of 93% and a specificity of 95%. The ROC curve for this model is shown in Fig. 4(a) and it is associated with an AUC of 0.98. These results were achieved using a model trained using only two spectral features, namely the peaks mostly associated with C–C stretching of proteins around $940\;{\mathrm{cm}}^{-1}$ and the symmetric aromatic ring breathing at $1004\;{\mathrm{cm}}^{-1}$ that is associated with the presence of phenylalanine. A classification model (Model B) was also trained to distinguish cancer from normal spectra from 145 measurements (87 cancer spectra and 58 normal spectra). This model had an accuracy of 91%, a sensitivity of 92%, a specificity of 90%, and an AUC of 0.96 based on four spectral features [Fig. 4(b)]. Those features corresponded to the same features as Model A, plus a peak associated with C–C skeletal stretching from proteins, lipids, and carbohydrates ($1129\;{\mathrm{cm}}^{-1}$) and a band around $1157\;{\mathrm{cm}}^{-1}$ associated with C-C/C-N skeletal stretching from proteins. Model C was developed to detect cancer from fat based on 180 Raman measurements (87 cancer spectra and 93 fat spectra). It performed with an accuracy of 98%, a sensitivity of 97%, and a specificity of 98%, with an AUC of almost 1 [Fig. 4(c)]. This model required two spectral features, namely, the $1004\;{\mathrm{cm}}^{-1}$ peak and the ${\mathrm{CH}}_{2}/{\mathrm{CH}}_{3}$ twisting deformation band at 1301 to $1304\;{\mathrm{cm}}^{-1}$.

**Fig. 5:**
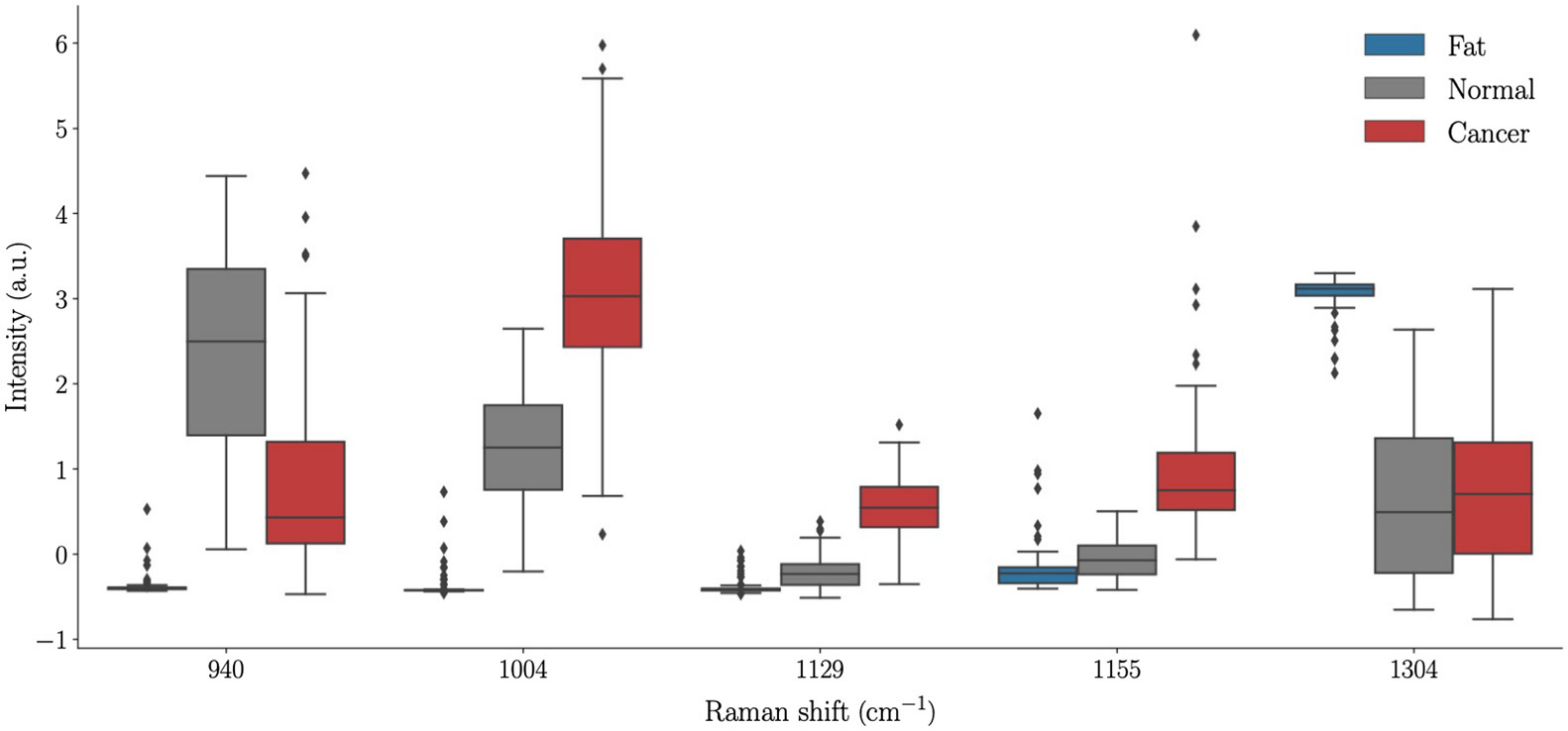
Statistical distribution of the intensity of the five features used by classification models for fat, normal and cancer spectra. Model A (cancer versus normal + fat) used features associated with bands at 940 and $1004\;{\mathrm{cm}}^{-1}$; Model B (cancer versus normal) used bands at 940, 1004, 1129, and $1155\;{\mathrm{cm}}^{-1}$; and Model C (cancer versus fat) used bands at 1004 and $1304\;{\mathrm{cm}}^{-1}$. Outliers are represented by black diamonds.

The spectral features that were used by the machine learning models are given in Table 2 along with their associated vibrational bonds and a tentative assignment with biomolecules based on the literature. Figure 5 shows the statistical distribution of these bands in the form of boxplots. The median (second quartile, Q2), first quartile (Q1), and third quartile (Q3) were clearly distinct for the two features used in model A (940 and $1004\;{\mathrm{cm}}^{-1}$). Q1, Q2, and Q3 were also well separated for cancer and normal tissue features associated with model B (940, 1004, 1129, and $1155\;{\mathrm{cm}}^{-1}$) as well as for cancer and fat for the features used in model C (1004 and $1304\;{\mathrm{cm}}^{-1}$).

**Fig. 6:**
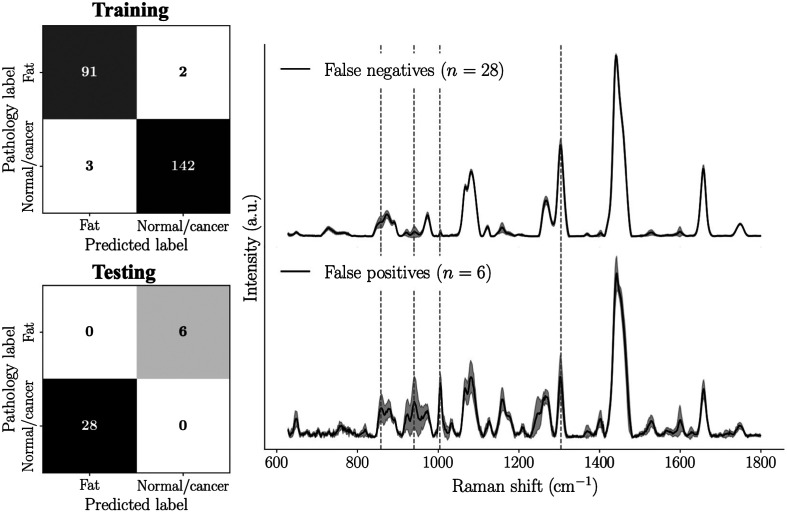
A *Fat* detection classifier was trained from 238 spectra. It was applied on the 34 spectra that were excluded from the study because of suspected mis-labeling likely associated with spatial registration errors of Raman spectroscopy measurements with the H&E images: the 28 spectra that were labeled as normal or cancer were all classified as fat (false negatives) and the six spectra that were labeled as fat were classified as normal or cancer (false positives).

A fourth model was trained to distinguish fat from all other tissue types (normal + cancer) from 238 spectra (Fig. 6), resulting in an almost perfect AUC of 1 with specificity and sensitivity >98%. This model was used to test whether the exclusion criteria leading to the rejection of suspected mis-labeled samples (28 labeled as normal or cancer, 6 spectra labeled as fat) was justified. The model was directly applied to those 34 spectra and predicted, in all cases, that the histology labels were inconsistent with the measured Raman signature. Specifically, the model predicted that all 28 normal or cancer spectra were actually fat, and that all 6 fat spectra were actually normal or cancer.

**Fig. 7:**
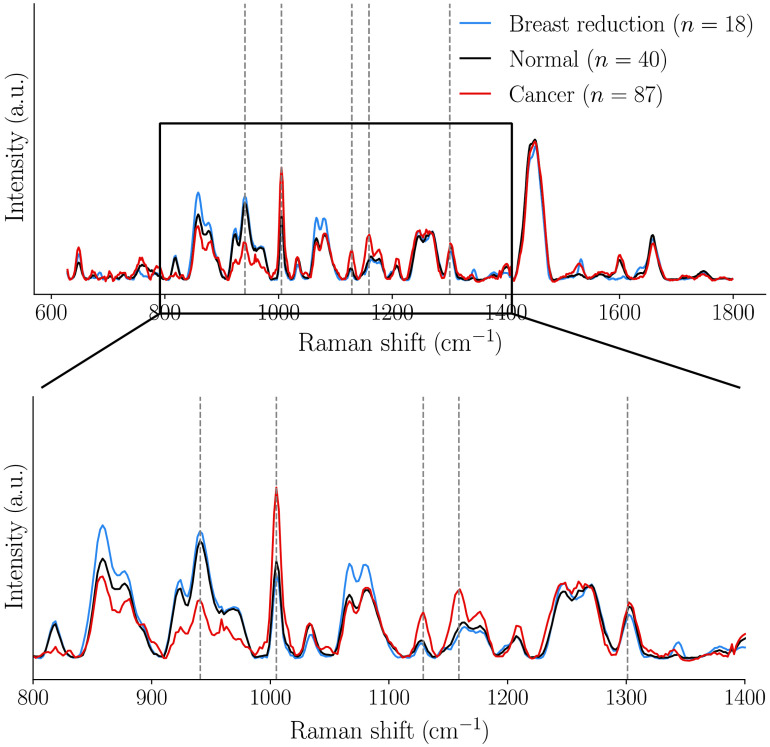
Raman spectra associated with the normal category from the breast reduction surgery patient, compared to all other normal measurements. Cancer spectra are also shown for visual comparison. The four features used in the classification models are identified with dotted lines.

In this study, more than 30% of all normal measurements were associated with the breast conserving surgery patient, which could have led to classification biases. However, Fig. 7 qualitatively shows that the average normal spectrum from the breast conserving surgery patient (n=18 spectra) was similar to the average spectrum associated to all other normal spectra in the dataset (n=40). More importantly, the average spectra associated with the normal category matched perfectly for the Raman bands used to train Models A, B, and C (Table 2), providing strong evidence no bias resulted from including a larger proportion of normal measurements from the same patient.

## 4 Discussion

This work demonstrated that it is possible to develop low complexity machine learning models with the ability to distinguish invasive breast cancer from healthy tissue. Here, the term low complexity means that all machine learning models were trained using only a few spectral features, each associated with known biomolecular vibrational modes. The resulting models are thus easily interpretable, a trait that could lower barriers to clinical adoption. This is important since the use of a small number of features reduces the burden imposed by the requirement for large datasets, a common issue encountered in biomedical applications of machine learning. Indeed, the confidence in estimated model performances achieved through cross-validation approaches is always higher when the number of features used during the model training phase is significantly smaller when compared to the number of training instances. In this work, 238 measurements from 20 patients were available for predictive modeling, and <5 spectral bands were used. This meant that the number of data types used for learning was lower than 5%, therefore much smaller than rule-of-thumb values recommended in machine learning, i.e., usually around 20% to 25%.

Another important aspect of this work was that significant efforts were made to ensure the trends associated with all retained Raman spectral features reflected—with high confidence—the actual nature of the tissue. This was achieved by ensuring model training and validation were done only using Raman measurements associated with high spectral quality and for which the spatial registration with histology analysis could be trusted. In fact, data selection was done to mitigate the limited reliability associated with the gold standard of histology used to assign labels to each measurement.

For those data points excluded from the machine learning model development process, the registration of the Raman measurements with the H&E-stained images resulted in labeling errors when assigning spectra to either the cancer, normal, or fat category. One cause of error was the change in shape between the specimen photograph and the H&E-stained image of the corresponding tissue section. The samples were compressed and rotated before being processed and stained, which sometimes made superposing the photograph with the H&E images difficult. Also, the specimen thickness was 1 cm or more and was sectioned in a 3 to 5  μm section before staining. Therefore, the surface on which the Raman measurements were acquired could be different than that located on the stained tissue sections. This source of inaccuracy was mitigated by having the location of each measurement marked on the H&E-stained image by a 3-mm diameter area that was significantly larger than the tissue area sampled with the Raman spectroscopy probe. Data points where the number of cells from the same category was too low (cancer: <80%, normal: <80%, and fat: <70%) within this 3-mm diameter area were also excluded. A more reliable spatial registration method between the gold standard of histology and the measurements would highly reduce the number of non-usable spectra that needed to be excluded in this study.

Using well-justified data point rejection criteria led to a smaller dataset, albeit associated with spectra having low inter-patient and intra-specimen variance. This allowed a clear biomolecular interpretation of the key Raman-predicted biomolecular differentiators between healthy and cancer tissue. The only feature present in all three models was the $1004\;{\mathrm{cm}}^{-1}$ peak corresponding to the aromatic amino acid phenylalanine. Other groups such as Zuniga et al. also obtained a sharp peak at the same Raman shift in breast cancer tissue.^44^ Compared to normal and adipose tissues, cancerous tissues had an increase of intensity at the phenylalanine peak. This was also confirmed by the increase of the peak at $1600\;{\mathrm{cm}}^{-1}$, which could also be associated with phenylalanine [Fig. 4(b)]. Gebrekidan et al. and Contorno et al. also found similar results of higher content in phenylalanine in breast tumor tissues.^45^^,^^46^ They hypothesized that this increase could be caused by the fact phenylalanine is the precursor of substances with mutagenic, genotoxic, and carcinogenic properties and that overexpression may be suggestive of the cancer pathogenesis.

The DNA/RNA peaks at 785, 1176, $1208\;{\mathrm{cm}}^{-1}$, and the proteins peaks at 760 and $1159\;{\mathrm{cm}}^{-1}$ were higher in breast tumor tissue. Many studies have confirmed genetic alterations in breast cancer disease.^47^^,^^48^ Specifically, the peaks at 785 and $1176\;{\mathrm{cm}}^{-1}$ correspond to vibrational modes of cytosine and several studies have found the implication of spontaneous and enzyme-catalyzed deamination of DNA cytosine bases.^49^ Similar findings of enhanced peaks of Amide III (peaks at 1246 and $1266\;{\mathrm{cm}}^{-1}$) have been reported in a study by Liu et al.^50^ Interestingly, Bitar et al.^51^ found that there were differences in the secondary structure of proteins of breast tissues that could differentiate subtypes of malignant lesions. In addition, they found that collagen content could be used to quantify the degree of pathogenicity of breast cancer.

The two peaks at 1129 and $1301\;{\mathrm{cm}}^{-1}$ corresponding to lipids were also enhanced in the breast cancer tissues compared to normal breast tissues. However, during the past years, our laboratory work in other pathologies has shown that most tumors have a lower intensity for peaks associated with lipids compared to normal tissues.^32^^,^^52^ Lipid metabolism in tumor cells has been widely studied, their biosynthesis is tightly related to signaling pathway. Li et al. found a decrease in lipids and suggested that tumors share a common phenotype of uncontrolled cell proliferation and, therefore, they consume large amounts of lipids.^53^ However, more recent studies have found a specific type of increase in lipids in breast tumor tissues: the unsaturated fatty acid. In 2016, Sixian You et al. observed an increase of polyunsaturated fatty acids in the human breast cancer tissues.^54^ Brozek-Pluska et al. (2019) also confirms the poly unsaturated fatty acid important increase in these malignant tissues.^55^ They have cautioned that disturbed ratio content of unsaturated and saturated lipids seems to be the marker of pathogenesis and not only the increase of polysaturated fatty acid. Further investigations would be needed to determine the different Raman spectra of polyunsaturated and monosaturated fatty acids composition in breast cancer.

Another tentative hypothesis is that while the band at $1129\;{\mathrm{cm}}^{-1}$ is solely associated with lipids, the band of $1128\;{\mathrm{cm}}^{-1}$ is however assigned to C─N stretching (proteins), C─O stretching (carbohydrates), and lipid transconformation. The contribution of proteins in cancer tissues adding to the presence of carbohydrates of the $1128\;{\mathrm{cm}}^{-1}$ band might have contributed to the upshifting of $1129\;{\mathrm{cm}}^{-1}$. All in all, lipids band assignments should be taken cautiously in breast cancer tissues. While there are contradictory findings about the increase or decrease of lipids in literature and among Raman spectroscopy work in breast cancer, the future work should be focusing on clarifying the roles of the lipids contribution in its malignancy. One way to do it would be to study the lipid phenotype of breast cancer cells and its metastatic spread. For example, Nieva et al.^56^ reported in 2014 the ability of Raman micro-spectroscopy to achieve rapid lipid profiling of breast cancer cells and therefore, distinguish metastatic ability from malignancy.

The intended use of the Raman spectroscopy system presented in this study is live surgical guidance during breast-conserving surgery. However, the total measurement times that were reported ranged from 1 to 40 s, for N=10 repeat measurements. Such large tissue interrogation times could impact practical use during surgical procedures. However, the data pre-processing and machine learning workflow used to produce the predictive models were re-applied on the subset of measurements associated with N=5 and N=1 accumulations, to assess potential degradation in performance associated with limited interrogation times. Interestingly, the accuracy and AUC of the ROC curve for those new models were essentially unchanged for N=5, and they were associated with limited changes for N=1. For example, for N=1 the accuracy and AUC of the cancer versus normal model was 88% and 0.89, compared to 91% and 0.97 for N=10. This implies that high levels of predictive accuracy could still be reached when lowering the range of measurement times to 0.1 to 4 s rather than 1 to 40 s.

Other practical concerns relate with the maximal permissible exposure (MPE) to tissue as set by ANSI standards for research laboratory laser safety.^57^ As an example, for a total light exposure duration of 1 s, MPE for skin is $0.97\;W/{\mathrm{cm}}^{2}$; for total duration >10  s, as was the case for the highest total integration time of 40 s, the MPE for skin is $0.3\;W/{\mathrm{cm}}^{2}$. In comparison, the measurements in this work led to energy densities of $1.04\;W/{\mathrm{cm}}^{2}$ when computed for hazard evaluation (i.e., 3.5-mm diameter limiting aperture) based on the rules set forth in ANSI Z136.1-2014 American National Standard for Safe Use of Lasers. It should be noted that those skin MPE values do not correspond to absolute thresholds beyond which damage to tissue will necessarily result in breast tissue. For example, none of the specimens in which Raman spectroscopy measurements were made presented morphological alterations (as assessed based on histology analyses) suggestive of heat-generated tissue damage. Future clinical applications using Raman spectroscopy in breast-conserving surgery will require detailed analyses to be done to determine acceptable photo-diagnostic levels, which will likely be significantly larger than the current MPE values set for skin.

Although the results presented here are very promising, work needs to be done to bring Raman spectroscopy in operating rooms for breast-conserving surgery guidance. For example, single-point measurements may not provide information sufficiently rapidly for surgeons to influence intraoperative decisions. This is because inspection of surgical cavities with large surface areas are expected in most cases, making successive single points of millimeter-sized areas unlikely to make the approach clinically deployable. To solve this problem, our group developed a handheld macroscopic Raman spectroscopy imaging instrument, specifically adapted for surgical guidance applications in breast oncology^58^^,^^59^ This system demonstrated hyperspectral detection of the fingerprint Raman signal over an area of $1\;{\mathrm{cm}}^{2}$, and classification model transferability across different biomedical Raman spectroscopy instruments was demonstrated, included with the Raman imaging system and the single-point probes.^32^ In the future, the classification models built in this study will therefore be used with the Raman imaging system for further studies in breast cancer.^59^

## 5 Appendix: Supplemental Material

Table 3 presented clinical information such as age, type of surgery, and type of cancer for every patient included in the study. It also shows the number of measurements taken on each class of tissues for each patient.

**Fig. 8:**
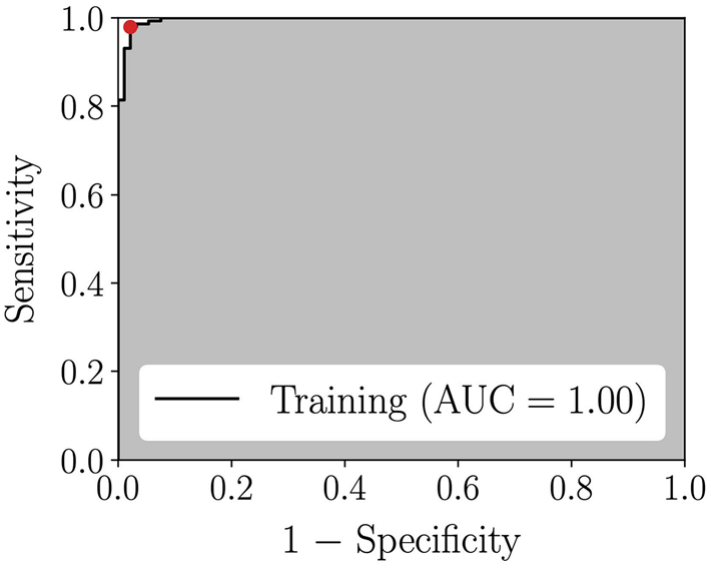
ROC curve for the fat detection model.

**Table 3:** Demographic and clinical information of the 20 patients included in the study.

Age	Surgery type	Tumor size (cm)	Invasive cancer type	NoS	Tissue type (before data selection)
55	Partial mastectomy	2.1	Ductal	3	Fat (7) cancer (6)
52	Partial mastectomy	1.2	Ductal	2	Fat (8)
82	Partial mastectomy	2.1	Ductal	2	Fat (8) cancer (14)
77	Partial mastectomy	2.2	Ductal	1	Fat (2) cancer (16) normal (4)
67	Partial mastectomy	2.2	Mammary	3	Fat (8) cancer (14)
77	Mastectomy	4 and 1	Lobular	2	Fat (6) cancer (8)
60	Lumpectomy	2.8	Ductal	3	Fat (1) cancer (8) normal (8)
50	Mastectomy	3 and 2	Ductal	3	Fat (15) cancer (16) normal (10)
60	Lumpectomy	1.6	Ductal	2	Fat (6) cancer (7)
67	Mastectomy	1	Ductal	1	Fat (1) cancer (2) normal (2)
61	Lumpectomy	NA	NA	NA	Fat (12) cancer (10)
76	Needle localized lumpectomy	1.3	Ductal	2	Cancer (6)
56	Mastectomy	NA	Mammary	2	Fat (1) cancer (11) normal (9)
78	Mastectomy	2 and 1.5	Lobular	2	Fat (4) cancer (5) normal (1)
67	Segmental mastectomy	1.1	Ductal	2	Fat (6) cancer (3)
68	Segmental mastectomy	3.2	Ductal	3	Fat (5) cancer (9)
70	Lumpectomy	3	Ductal	2	Fat (3) cancer (5) normal (25)
58	Lumpectomy	2.2	Lobular	3	Fat (4) cancer (2)
54	Lumpectomy	1 cm	Ductal	2	Fat (3) cancer (5) normal (1)
NA	Breast reduction	Absent	Absent	NA	Fat (2) normal (20)

## Code, Data, and Materials Availability

The data and materials information that support the findings of this study are available from the corresponding author upon reasonable request.
